# Glenoid inclination in reverse total shoulder arthroplasty with and without baseplate wedge augmentation: a retrospective comparative study

**DOI:** 10.1016/j.jsea.2026.100090

**Published:** 2026-09-09

**Authors:** Riki D. Wylie, Clement Chan, Jay R. Ebert, Travis M. Falconer

**Affiliations:** a Sir Charles Gairdner Hospital, Perth, Australia; b School of Medicine, The University of Western Australia, Perth, Australia; c School of Human Sciences (Exercise and Sport Science), The University of Western Australia, Perth, Australia; d Perth Orthopaedic & Sports Medicine Centre, Perth, Australia

**Keywords:** Glenoid inclination, Reverse total shoulder arthroplasty, Shoulder, Augment, Augmented baseplate, Wedge, Standard baseplate

## Abstract

**Background:**

Superior inclination of the glenoid component in reverse shoulder arthroplasty (rTSA) is avoided due to the associated risks of impaired range of motion, scapular notching, glenoid loosening, and instability. Metallic wedge baseplates have been explored as an augment to rTSA that corrects pre-operative superior angulation while preserving native bone. The aim of this study was to compare glenoid inclination between rTSAs with standard and wedge augmented baseplates.

**Methods:**

This was a single-center retrospective comparative study. Two independent reviewers assessed de-identified shoulder x-rays and computed tomography (CT) images for all patients who underwent rTSA by a single surgeon from June 2022 to June 2025. Patients received either an Aequalis Reversed II standard baseplate or a Tornier Perform 15° full wedge augmented baseplate. Pre-operative inclination was measured using total shoulder arthroplasty (TSA) angle, rTSA angle, and Stryker Blueprint. Post-operative inclination was measured with the rTSA angle on x-ray to assess correction.

**Results:**

The study included 106 shoulders from 103 patients who underwent rTSA (49 standard, 57 wedge). For inter-rater reliability, intraclass correlation coefficients were 0.911 and 0.905 for the pre-operative x-ray and CT TSA angles and 0.931 and 0.844 for the pre-operative x-ray and CT rTSA angles. A significant and large correlation (r = 0.64, *P* < .001) was observed between pre-operative Stryker Blueprint™ and measured TSA angles and between x-ray and CT methods in assessment of pre-operative TSA (r = 0.62, *P* < .001) and rTSA (r = 0.58, *P* < .001) angles. Overall, a significant reduction (*P* < .001) in the rTSA angle was observed from pre- to post-surgery (−20^°^ ± 10^°^ [standard −24° ± 10°; wedge −15° ± 8^°^]). There was no pre-operative difference (*P* = .180) in the rTSA angle between the standard and wedge groups; however, the standard group demonstrated a significantly lower (*P* < .001) rTSA angle post-operatively (standard −6^°^ ± 8; wedge 1^°^ ± 8).

**Conclusion:**

Standard baseplates were shown to have a significantly lower rTSA angle post-operatively when compared to the Tornier Perform 15^°^ full wedge augmented baseplates. Both standard and wedge augmented baseplates significantly reduced superior glenoid inclination.

The use of reverse total shoulder arthroplasty (rTSA) continues to increase in prevalence, with indications expanding to include end-stage osteoarthritis, rotator cuff arthropathy, and proximal humerus fractures.^16^^,^^26^^,^^41^^,^^50^^,^^53^^,^^58^ Recent Australian Joint Registry data demonstrate increasing popularity of rTSAs, with the majority of total shoulder arthroplasties (TSAs) now stemmed rTSAs.^38^ During operative planning for rTSA, superior inclination of the glenoid component is avoided. This is due to its association with an increased risk of scapular notching, glenoid instability, aseptic glenoid baseplate loosening, and impaired range of motion.^14^^,^^15^^,^^17^^,^^18^^,^^34^^,^^35^^,^^39^^,^^48^ Although optimal coronal positioning is debated, neutral or inferior angulation up to −10° is accepted.^12^^,^^23^, ^24^, ^25^^,^^45^^,^^54^

Several methods have been employed to correct glenoid deformities and achieve satisfactory glenoid inclination in rTSA. Eccentric reaming of the glenoid has historically been used to correct angulation when using a standard flat glenoid baseplate, though it is associated with the risk of excessive medialization and glenoid bone stock loss.^1^^,^^7^^,^^8^^,^^19^ Both bone loss and excessive medialization increase the risk of scapular notching, imbalance of soft tissue tensioning of the rotator cuff and deltoid, instability, and implant failure.^3^^,^^5^^,^^6^^,^^11^^,^^20^^,^^34^^,^^44^^,^^59^ This is particularly true with superior erosion patterns (Favard E2) common to cuff tear arthropathy, due to chronic superior humeral head migration.^52^ Augmentation of the baseplate has thus been explored to correct defects in the glenoid and address deformity without removing bone stock and medializing the joint line. This includes bone grafting, or bony increased-offset rTSA (BIO-rTSA), as well as metallic wedged baseplates. BIO-rTSA, however, has been associated with risk of failure from bone graft resorption, glenoid loosening, and non-union.^10^^,^^13^^,^^27^, ^28^, ^29^^,^^40^^,^^57^

Metallic wedge baseplates have therefore been trialed as an alternative that allows for less bone reaming, without the risks carried by BIO-rTSA. In a computed tomography (CT)-based software study by Shah et al^51^, the use of 10^°^ metallic augmented baseplates, when compared to standard baseplates, reduced estimated bone removal by approximately 50% and resulted in 2.4 mm less medialization. Existing literature suggests metallic wedge baseplates have comparable range of motion, patient-reported outcomes, and complication and revision rates when compared with BIO-rTSA^28^^,^^32^^,^^42^^,^^55^ and standard baseplates.^22^^,^^33^^,^^36^^,^^37^^,^^43^^,^^46^^,^^49^^,^^56^ Few studies, however, have compared the correction of glenoid inclination from pre- to post-surgery between standard and wedge augmented rTSA baseplates. The primary aim of the study was thus to determine whether a 15^°^ metallic wedge augment can correct glenoid inclination in an anatomic manner comparable to a standard baseplate. The primary hypothesis was that if a wedge baseplate was used instead of a standard baseplate, there would be no difference in post-operative inclination.

## Methods

### Study design and patient selection

This was a single-center retrospective comparative study of glenoid inclination pre- and post-rTSA with wedge or standard baseplates. Two independent reviewers assessed de-identified shoulder x-rays and CT images for all consecutive patients ≥18 years of age who underwent rTSA by a single fellowship-trained surgeon from June 2022 to June 2025. This period captured the change in the routine practice of the surgeon from using standard baseplates to 15^°^ full wedge augmented baseplates for all patients receiving rTSA, irrespective of morphology. Patients were grouped based on baseplate type into standard or wedge augmented. Patients were excluded if the surgery was a revision procedure, involved a BIO-rTSA, lateralized baseplate, or custom prosthetic, or if there was inadequate imaging, or no pre-operative x-ray or CT available. All patients had post-operative x-rays taken the day following surgery as part of routine practice. These radiographs were used to assess correction in glenoid inclination.

### Implants

Patients in the standard baseplate group received the Aequalis Reversed II baseplate (Tornier Inc, Memphis, USA). All patients included in the augmented wedge group received 15^°^ full wedge baseplates from the Tornier Perform Reverse Shoulder Arthroplasty system (Stryker, Michigan, USA). All implants used cobalt-chrome glenospheres with diameters of 36 mm, 39 mm, or 42 mm. Porous titanium coating stems were used with standard length, 132.5^°^ inclination, and varying sizes to fit patient anatomy. Tray offset was either 1.5 mm or 3.5 mm, and 0 mm thickness. Standard inserts with 6 mm or 9 mm thickness and −12.5° tilt were used.

### Surgical technique

All procedures were performed by a single fellowship-trained shoulder surgeon using a standard deltopectoral approach. Pre-operative planning was undertaken using CT, with 3-dimensional assessment performed using Stryker Blueprint software to evaluate glenoid morphology and inclination. Glenoid preparation and baseplate implantation were undertaken using conventional instrumentation without the use of patient-specific instrumentation, navigation, or robotic assistance.

Following exposure, the long head of the biceps was routinely managed with tenodesis. The subscapularis tendon was released via tenotomy to allow exposure of the glenohumeral joint and glenoid. At the conclusion of the procedure, the subscapularis was repaired using non-absorbable number 5 sutures passed through drill holes in the lesser tuberosity and tied around the stem of the prosthesis, according to standard technique.

For standard flat baseplates, glenoid preparation was performed using established eccentric reaming techniques, preferentially reaming the inferior glenoid to correct superior inclination while preserving subchondral bone. A standard Stryker glenoid guide was used, with the guide pin placed through the 10° inferior inclination hole to assist with correction of superior tilt. The aim was to achieve a final glenoid inclination of neutral to approximately 10^°^ of inferior tilt, while avoiding excessive medialization (humeral axis >15 mm medial to lateral border of acromion). Within the literature there is no clearly defined ideal amount of medialization or lateralization; however, preservation of bone stock and restoration of native tension of the remaining cuff and soft tissues was a primary operative goal. Where eccentric reaming resulted in a superior gap between the baseplate and native glenoid, structural bone graft harvested from the resected humeral head was used to fill the defect. In cases where the residual gap was minimal, the baseplate was intentionally seated 1–2 mm off the native glenoid surface. There was no maximal depth of reaming set for this process; however, it was routinely 2-3 mm of native bone to achieve the desired inferior inclination. With this technique a minimum area of back side contact for the baseplate of 50% was accepted to achieve the desired inclination. This was planned on the pre-operative CT scan, and the maximal depth was often in the inferior 50% of the native glenoid.

For the 15° wedge augmented baseplates, the standard wedge baseplate guide supplied within the Stryker instrumentation set was used. This guide incorporates a 15° angled reaming surface with a slightly spherical posterior contour. The guide was positioned to match the concavity of the native or arthritic glenoid and rotated to accommodate the dominant deformity pattern. It was typically seated as inferiorly as possible, with the apex of the wedge oriented superiorly or posterosuperiorly, reflecting the most common patterns of superior glenoid wear. This technique allowed correction of superior glenoid inclination toward neutral alignment, and in some cases to slight inferior inclination, while minimizing glenoid bone removal.

In both techniques, baseplate positioning aimed to optimize inferior placement and inclination correction while maintaining fixation in native bone. Final baseplate fixation and glenosphere implantation were performed according to standard manufacturer recommendations.

### Assessment

Demographic data were obtained, including age, sex, operated shoulder, and primary diagnosis. Favard and Walch classifications for each glenoid, as assessed by the fellowship-trained surgeon, were recorded. Pre-operative shoulder inclination was measured on x-ray and CT with both reverse shoulder arthroplasty (rTSA) and TSA angles.^4^ The rTSA angle is defined as the angle between the inferior part of the glenoid fossa and the perpendicular to the floor of the supraspinatus (Fig. 1, *A*), whereas the TSA angle is between the global glenoid fossa and the perpendicular to the floor of the supraspinatus fossa (Fig. 1, *B*). For those who had available CT and pre-operative planning using Stryker Blueprint, TSA angles derived from this software were recorded. Post-operative inclination was measured by the rTSA angle on x-ray, using the baseplate as the glenoid portion.

**Figure 1:**
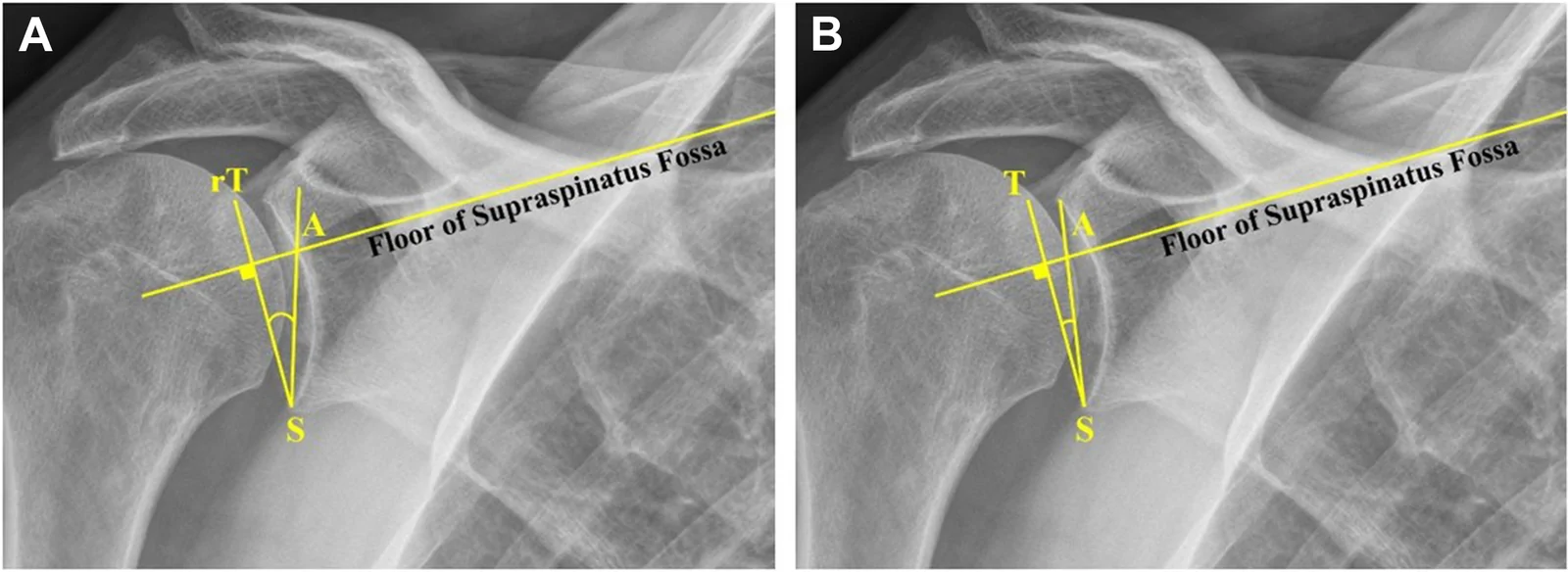
Images demonstrating radiographic assessment of inclination: (**A**) rTSA angle, (**B**) TSA angle. *TSA*, total shoulder arthroplasty; *rTSA*, reverse shoulder arthroplasty.

### Data and statistical analysis

Continuous variables were reported as means (ranges), and categorical variables as frequencies (percentages) where relevant. Independent sample *t*-tests and chi-squared tests were undertaken to compare baseline demographic and glenoid morphology between the wedge-augmented and standard baseplate patient cohorts.

Interobserver reliability was assessed between 2 scorers using intraclass correlation coefficients (ICCs) for the pre-operative TSA and rTSA angles on x-ray and CT. ICCs were reported as poor (<0.5), moderate (0.5-0.75), good (0.75-0.9), or excellent (>0.9).^31^

Pearson's correlation coefficients were used to determine the significance and strength of the association between: (1) the pre-operative Stryker Blueprint and the pre-operative TSA angles assessed, and (2) the pre-operative x-ray and CT scores (TSA and rTSA angles). The strength of associations was categorized according to Cohen's criteria as follows: greater than 0.5 (large), 0.3-0.5 (moderate), 0.1-0.3 (small), and less than 0.1 (trivial).^9^

Repeated measures analysis of variance evaluated the change in rTSA angles from pre-to post-surgery, between the wedge and standard groups. Otherwise, independent sample *t*-tests were undertaken to compare pre-operative inclination measures between the wedge and standard baseplate groups.

Statistical significance was determined at *P* < .05, with analysis performed using SPSS (SPSS, Version 30.0, SPSS Inc., USA). A total minimum sample of 84 patients was required (42 per group, standard and wedge baseplate) to detect a large effect size of 5° with 90% power and an alpha of 0.05.

To account for multiple testing, Benjamini-Hochberg correction was applied to control the false discovery rate at q < 0.05.^2^

## Results

A total of 141 shoulders were identified who underwent rTSA over the nominated time period. Overall, 35 patients were excluded for reasons including no pre-operative x-ray or CT (n = 17), poor radiographic views (n = 12), fully lateralized baseplates (n = 3), BIO-rTSAs (n = 2), and revision surgery (n = 1). A total of 106 shoulders (49 standard, 57 wedge) in 103 patients were included in the final analysis (Fig. 2). No group-based (wedge versus standard baseplate) differences were observed for age (*P* = .374), sex (*P* = .617), operated shoulder (*P* = .560), or the distribution of Walch (*P* = .627) or Favard (*P* = .989) glenoid morphology, though a significant difference was seen for primary diagnosis (*P* < .001) (Table I).

**Figure 2:**
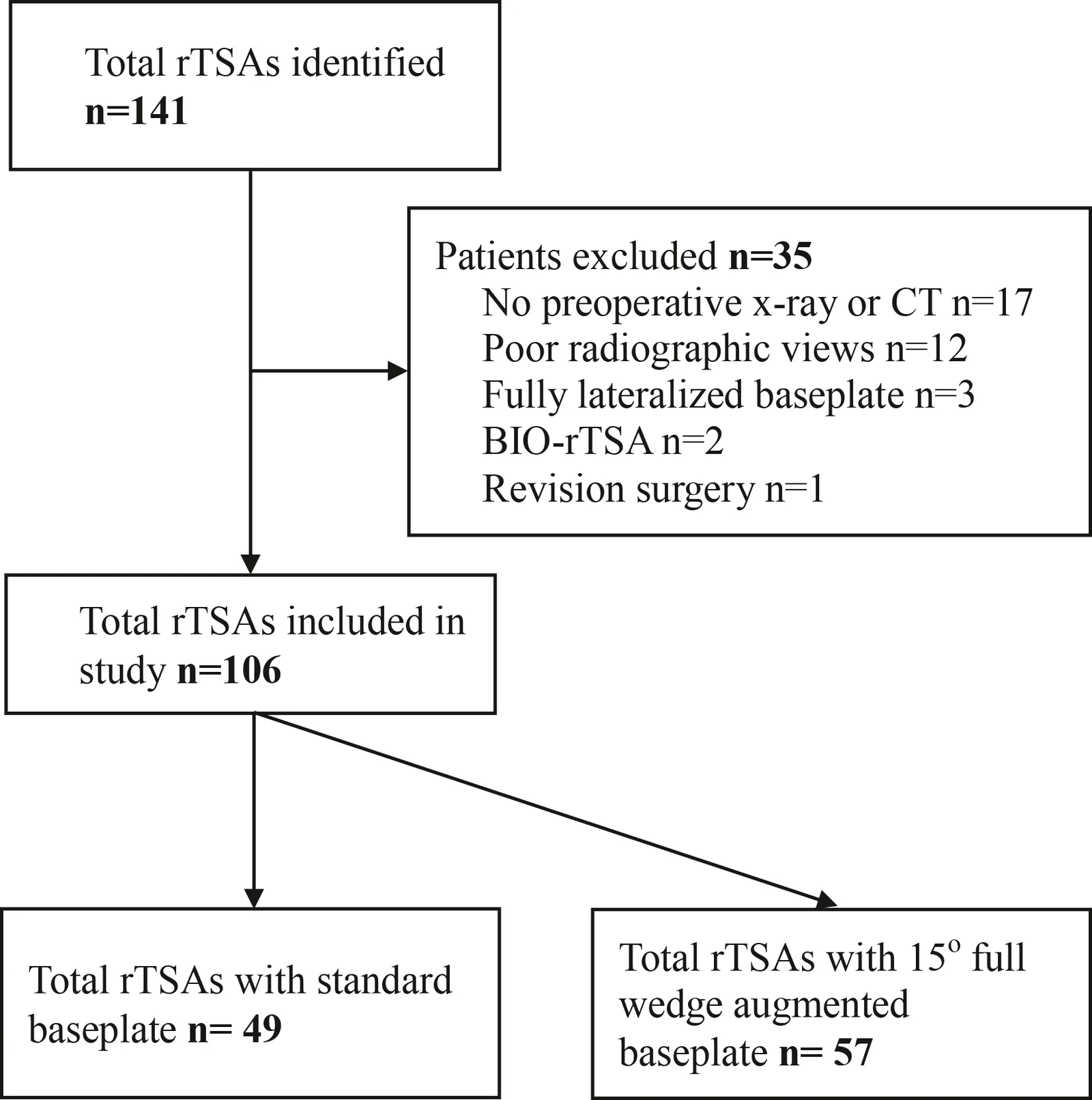
Study sample flowchart. *rTSA*, reverse shoulder arthroplasty; *CT*, computed tomography; *BIO-rTSA*, bony increased-offset rTSA.

**Table I:** Patient demographics

Characteristic	Standard (n = 49)	Full wedge (n = 57)	Total (n = 106)	*P* value
Age at procedure, yr, mean ± SD (range)	74.7 ± 7.97 (58-94)	73.3 ± 8.12 (51-88)	73.9 ± 8.07 (51-94)	.374
Sex				.617
Female	25 (51%)	33 (58%)	58 (55%)	
Male	24 (49%)	24 (42%)	48 (45%)	
Operated shoulder				.560
Left	19 (38%)	19 (33%)	38 (36%)	
Right	30 (61%)	38 (67%)	68 (64%)	
Primary diagnosis				<.001∗
Cuff tear arthropathy	25 (51%)	15 (26%)	40 (38%)	
Humerus fracture	8 (16%)	1 (2%)	9 (8%)	
Massive rotator cuff tear	4 (8%)	32 (56%)	36 (34%)	
Post-traumatic arthritis	3 (6%)	0 (0%)	3 (3%)	
Primary glenohumeral osteoarthritis	9 (18%)	9 (16%)	18 (17%)	
Favard classification				.989
E0	34 (69%)	39 (68%)	73 (69%)	
E1	10 (20%)	12 (21%)	22 (21%)	
E2	3 (6%)	3 (5%)	6 (6%)	
E3	2 (4%)	3 (5%)	5 (5%)	
E4	0 (0%)	0 (0%)	0 (0%)	
Walch classification				.627
A1	26 (53%)	30 (53%)	56 (53%)	
A2	10 (20%)	9 (16%)	19 (18%)	
B1	6 (12%)	11 (19%)	17 (16%)	
B2	5 (10%)	6 (11%)	11 (10%)	
B3	0 (0%)	0 (0%)	0 (0%)	
C	2 (4%)	1 (2%)	3 (3%)	
D	0 (0%)	0 (0%)	0 (0%)	

*SD*, standard deviation.

All percentages are reported to the nearest integer value.

∗ Denotes a significant P value less than 0.05.

For the inter-rater reliability analysis, ICCs were 0.911 and 0.905 for the pre-operative x-ray and CT TSA scores, respectively, and 0.931 and 0.844 for the pre-operative x-ray and CT rTSA scores, respectively. This indicated good-excellent reliability between the 2 observers.

A significant and large correlation (r = 0.64, *P* < .001) was observed between the pre-operative Stryker Blueprint™ and pre-operative CT TSA angles measured. Significant and large correlations were also observed between x-ray and CT methods in the assessment of pre-operative TSA (r = 0.62, *P* < .001) and rTSA (r = 0.58, *P* < .001) angles (Table II).

**Table II:** Difference in x-ray and CT in patients with both imaging modalities (n = 66)

Pre-operative inclination measure	X-ray Mean ± SD	CT Mean ± SD	CT – x-ray Mean ± SD	Correlation Strength, significance
Pre-op TSA angle (°)	9 ± 7	11 ± 7	2 ± 6	r = 0.62, *P* < .001
Pre-op rTSA angle (°)	17 ± 8	20 ± 8	2 ± 7	r = 0.58, *P* < .001

*SD*, standard deviation; *CT*, computed tomography; *TSA*, total shoulder arthroplasty; *rTSA*, reverse shoulder arthroplasty.

A significant reduction (*P* < .001) in the x-ray rTSA angle was observed from pre- to post-surgery. Although no analysis of variance group-based difference (*P* = .064) was observed, a significant interaction effect (*P* < .001) was observed. There was no pre-operative difference (*P* = .180) observed between the standard and wedge groups; however, the standard group demonstrated a significantly lower (*P* < .001) rTSA angle post-operatively (Table III). The standard baseplate had a correction of −24^°^ ± 10, whereas the full wedge augment was −15^°^ ± 8. The mean change in inclination was −19^°^ ± 10.

**Table III:** Glenoid inclination based on imaging modality available

Pre-operative x-ray (n = 86)			
Measurement	Standard (n = 44) Mean ± SD	Full wedge (n = 42) Mean ± SD	*P* value
Pre-op x-ray TSA angle (°)	10 ± 6	8 ± 7	.422
Pre-op x-ray rTSA angle (°)	20 ± 8	16 ± 9	.180
Post-op x-ray rTSA angle (°)	−6 ± 8	1 ± 8	<.001∗
Correction in rTSA angle (°)	−24 ± 10	−15 ± 8	<.001∗

Pre-operative CT (n = 86)			
Measurement	Standard (n = 33) Mean ± SD	Full wedge (n = 53) Mean ± SD	*P* value
Pre-op CT TSA angle (°)	11 ± 7	12 ± 7	.715
Pre-op CT rTSA angle (°)	19 ± 8	21 ± 8	.280
Pre-op Blueprint TSA angle (°)	8 ± 7	7 ± 6	.609
Post-op x-ray rTSA angle (°)	−5 ± 8	0 ± 8	.009∗

Pre-operative x-ray and CT (n = 66)			
Measurement	Standard (n = 28) Mean ± SD	Full wedge (n = 38) Mean ± SD	*P* value
Pre-op TSA angle (°)			
X-ray	10 ± 7	9 ± 7	.424
CT	11 ± 8	11 ± 7	.847
Blueprint^TM^	8 ± 7	7 ± 5	.409
Pre-op rTSA angle (°)			
X-ray	18 ± 8	17 ± 9	.555
CT	19 ± 8	20 ± 7	.618
Post-op X-ray rTSA angle (°)	−5 ± 9	1 ± 8	.007∗

Pre-operative Blueprint (n = 95)			
Measurement	Standard (n = 39) Mean ± SD	Full wedge (n = 56) Mean ± SD	*P* value
Blueprint TSA angle (°)	8 ± 7	7 ± 6	.846

Post-operative x-ray (n = 106)			
Measurement	Standard (n = 49) Mean ± SD	Full wedge (n = 57) Mean ± SD	*P* value
Post-op rTSA angle (°)	−6 ± 8	0 ± 8	<.001∗

*SD*, standard deviation; *CT*, computed tomography; *TSA*, total shoulder arthroplasty; *rTSA*, reverse shoulder arthroplasty.

∗ Denotes a significant *P* value less than 0.05.

There was no significant difference observed between standard and wedge groups in pre-operative CT TSA (*P* = .715) or rTSA (*P* = .280) angles (Table III). A significantly lower (*P* = .009) post-operative x-ray rTSA angle was observed in the standard compared to the wedge group.

For patients who had both pre-operative x-ray and CT available (n = 66), there was no significant difference observed between standard or wedge groups for pre-operative TSA angles (x-ray, CT, or Stryker Blueprint), or rTSA angles (x-ray or CT), respectively (Table III). The standard group had a significantly lower (*P* = .007) x-ray rTSA angle observed post-operatively compared to the wedge group.

There was no significant difference observed in Blueprint inclination between standard and wedge groups (*P* = .846) (Table III). Mean Blueprint inclination was 7^°^ ± 6. For the overall population, the mean post-operative rTSA angle was -2^°^ ± 9 (-6^°^ ± 8 for standard, 0^°^ ± 8 for full wedge).

All significant associations reported survived Benjamini-Hochberg correction for multiple comparisons.

## Discussion

The main finding of this study was that standard baseplates demonstrated a significantly lower rTSA angle post-operatively when compared to the wedge augmented baseplates. Both standard and 15^°^ wedge augmented baseplates, however, significantly reduced superior glenoid inclination.

Due to the risks associated with superior glenoid inclination in rTSA,^14^^,^^15^^,^^17^^,^^18^^,^^34^^,^^35^^,^^48^ it is important to establish that appropriate correction in angulation can be achieved with wedge augmented baseplates. Of interest, while both standard and wedge augmented baseplates significantly reduced superior glenoid inclination from pre- to post-surgery, the primary hypothesis was not supported in that standard baseplates demonstrated significantly lower rTSA angles post-operatively compared to 15^°^ wedge augmented baseplates. Despite this, on average the 15^°^ wedge achieved near neutral inclination irrespective of patient morphology. Significant correction in inclination was expected and in keeping with prior literature.^21^^,^^30^^,^^46^^,^^47^^,^^56^ Guehring et al^21^ demonstrated that mean rTSA angle could be corrected to near neutral with 360^°^ metal hemi-wedge baseplates for patients with glenoid deformity. Few studies, however, had previously compared post-operative inclination between standard and wedge-augmented baseplates. Verrall et al^56^ reported no difference in post-operative rTSA angle between a cohort that received standard baseplates and one that received SMR TT augmented 360 baseplates; however, mean baseplate tilt was superiorly inclined in both groups. The post-operative group-based difference in rTSA angle in the current study may be due to excessive eccentric reaming with standard baseplates to be more conservatively within the 0^°^ to −10^°^ range. Conversely, it may be due to an overestimate of the degree of correction attained when using the 15^°^ wedge baseplate, or a combination of both factors.

A secondary finding from this study, through comparison of rTSA and TSA angle measurements, was that the TSA angle underestimated pre-operative inclination relative to the rTSA angle. This is consistent with the original paper by Boileau et al^4^ that proposed the rTSA angle as a more accurate means of measuring glenoid inclination in rTSAs and noted the TSA angle was on average 9^°^ ± 7 and 10^°^ ± 5 lower than the mean rTSA angle with x-ray and CT, respectively. In the current study, x-ray correlated strongly with CT imaging for both TSA and rTSA angle measures.

This study is not without limitations, with the analysis firstly limited by its design as a retrospective observational study, inherently introducing risk of bias. It is also important to note that the cohorts in the study were mostly non-concurrent. During the study period the surgeon changed practice from the use of standard to wedge baseplates for all patients at baseline. Hence, the default selection of baseplate depended on the date of operation rather than the indication. There was, however, a period of overlap in use of baseplate types. While reducing selection bias, it is certainly not excluded. Not being parallel also introduces other confounders, such as the surgeon's skill or other aspects of routine practice changing over time. The temporal difference may explain the discrepancies seen in primary diagnosis between groups. The patients included in the study were predominantly diagnosed with either cuff tear arthropathy or massive rotator cuff tear, and most glenoid morphologies were Favard classification E0 or E1, Walch classification A1. Unfortunately, the study was underpowered to conduct subgroup analysis based on either primary diagnosis or glenoid morphology.

Measurement of inclination on x-ray was dependent on the projection and quality of the radiographs available. This included variations in radiographers, shoulder position, views, and patient anatomy. Ultimately, 12 participants were excluded due to inadequate x-rays; however, within those included, there was variability in the standard of x-rays, which may have impacted the accuracy of assessment. Not every patient had pre-operative CT, and post-operative CT was not a part of routine follow-up. The post-operative x-rays were all taken day one post-operatively. Therefore, we were unable to assess for long-term radiographic complications such as scapular notching or glenoid loosening, or whether the correction in inclination was maintained. Further, not all patients had both CT and x-ray available, preventing powered comparison of the 2 imaging modalities. As a radiographic study, no clinical correlation can be made to measured outcomes.

Although the surgeon utilized pre-operative planning, only standard instrumentation was used. This may impact the generalizability of the findings to surgery aided by patient-specific instrumentation, navigation, or robotic assistance. Furthermore, there exist large variations in implant designs used in practice. The study, however, assessed only 2 types of baseplates, limiting the application of findings to other implants. It should also be noted that one of the authors received a funding grant from Stryker Australia Pty Ltd for research assistant hours and radiology review costs.

Despite the limitations of this study, the results suggest that use of a 15° wedge baseplate can reliably achieve near-neutral glenoid inclination when applied to a population predominantly comprising cuff tear arthropathy and E0/E1 glenoid morphology. In our technique, a standard 15° guide is used and rotated to match the native glenoid morphology, allowing the reamer to seat concentrically on the glenoid surface and optimize backside contact, typically exceeding 80%-90%. This approach facilitates correction of superior inclination while minimizing bone removal and avoiding excessive medialization. When applied in this manner, metallic wedge baseplates appear to represent a safe and reproducible adjunct in rTSA, providing adequate inclination correction to mitigate known complications associated with superior tilt. Although the absolute degree of correction may be less than that achievable with standard baseplates combined with aggressive eccentric reaming, this technique offers a bone-preserving alternative with predictable alignment. Further large prospective studies are required, and should explore the degree of medialization between techniques, long-term comparative radiographic outcomes, including CT, and correlate these with functional outcomes and patient-reported outcomes, with stratification by glenoid morphology and underlying pathology.

## Conclusion

In rTSA, the use of the Aequalis Reversed II standard baseplate and the Tornier Perform 15^°^ full wedge augmented baseplate both resulted in significant deformity correction in the coronal plane. Standard baseplates may provide greater correction; however, both produce post-operative shoulder inclination within the accepted range.

## Disclaimers:

Funding: Stryker Australia Pty Ltd provided a research grant to fund the cost of non-rebated CT scans.

Conflicts of interest: Travis M. Falconer received a research grant obtained from Stryker Australia Pty Ltd, paid to Perth Orthopaedic & Sports Medicine Centre Research Institute. Stryker Australia Pty Ltd was not involved in data collection, data analysis, or the preparation of or editing of the manuscript. He also received consulting fees for paid presentations, teaching, and demonstrations—none of which relate to the research. Any additional authors, their immediate families, and any research foundations with which they are affiliated have not received any financial payments or other benefits from any commercial entity related to the subject of this article.
